# Personality and Social Context Factors Associated to Self-Reported Excessive Use of Information and Communication Technology (ICT) on a Sample of Spanish Adolescents

**DOI:** 10.3389/fpsyg.2019.00436

**Published:** 2019-03-04

**Authors:** Maria de las Mercedes Martín-Perpiñá, Ferran Viñas Poch, Sara Malo Cerrato

**Affiliations:** Department of Psychology, University of Girona, Girona, Spain

**Keywords:** adolescents, personality, social context, excessive ICT use, risk factors

## Abstract

The vulnerability that characterizes adolescents justifies the growing health concern about the impact of excessive use of ICT. Exploring the roll both psychological and social variables in excessive use of ICT in adolescents can help preventing risk behaviors. Examining the ICT use of 1,102 secondary school, baccalaureate and professional training course students (11–18 years old, *M* = 14.42, *Sd* = 1.78; 50.58% boys), we investigate the psycho-social profile of those adolescents who have self –reported an excessive use of ICTs. Personality factors were assessed using the NEO-FFI, and social context factors through the PWI (Personal Well-being Index); AF5 (Multidimensional Scale of Self Concept); SSA (Social Support Appraisals); *ad hoc* questions on self-perception of parental and sibling ICT consumption, and the existence of rules for ICT use in the home. 14.5% (*n* = 160) of adolescents match the criteria of excessive use. The self-reported excessive use is associated as much by personality factors as by family context. Risk factors in self-reported excessive adolescent ICT use are being female, impulsiveness, perceiving a high level of family support and a high use by parents and siblings. The family, academic, emotional and physical self-concepts are factors of protection. This study has provided us with a profile of adolescents who make a self-reported excessive use of ICT, which may be of help in preventing such use. The presence of these specific personality and socio-demographic factors indicate a greater vulnerability and may serve as indicators for parents, teachers and healthcare professionals to intervene and prevent excessive ICT use and other serious psychological problems related.

## Introduction

Adolescents who have been born and raised in the “Information Society” or the “Digital Era” are the age group that most frequently connects to the Internet and makes greatest use of mobile telephones; as such, they are the focus of an increasing amount of research (Sánchez-Carbonell et al., 2008; Batalla et al., 2012). While ICT use increases with age (Devís-Devís et al., 2009), an increasing number of studies highlight that this begins earlier and earlier, with massive use detected in pre-adolescence (Fernández-Montalvo et al., 2015). Even though adolescent ICT use is massive among both sex, according with Rial et al. (2015) girls use them more frequently and intensively than boys. Recent studies indicate significant differences by gender in the tendencies of use: in the case of girls, a more expressive-communal use related to social interactions, very remarkable in the closest social relationships, and shared in social networks through public channels, with more loaded contents of affectivity and more telling of their daily experiences; while men are more oriented toward an agent-instrumental, more action-related use, playing videogames in ICT applications and with more pragmatic tendencies in its contents. These findings might suggest that females tend, more than males, to prefer indirect communication. The social need more prominent in girls to be always connected to ICTs or Social Networks may suggest that gender can be a risk factor.

While there is no empirical evidence, it has been argued that females show an enhanced vulnerability to Social Networking addiction (Kuss and Griffiths, 2011). Toda et al. (2006) concluded that being female is a risk factor in ICT addiction.

Although there are insufficient criteria with which to diagnose addiction to ICTs, the excessive use of technology is recognized as a public health concern (World Health Organization, 2014). Numerous studies have aimed to calculate excessive use in specific population groups. One study of adolescents in 11 European countries found problematic Internet use to be 13.5% (Durkee et al., 2012); while Weinstein and Lejoyeux (2010) put the rate in the United States at between 0.3% and 25%. Data published in the Spanish Government’s Home Office *Survey of Internet Use and Safety* (Instituto Nacional de Estadística [INE], 2014) showed that 60% of children and young people said they connected to the Internet every day, mostly through mobiles and computers. A study carried out by the Fundación Mapfre (2014) concluded that 21.3% are at risk of developing Internet-addictive behavior through their use of social networks.

There is a lack of consensus among the scientific community as to whether excessive ICT use can be considered a similar kind of addictive disorder as that of behavioral addictions. American Psychiatric Association (2013) The authors of the DSM-V (2013) stated that such an addiction cannot yet be considered to exist, although they do include *Internet gaming disorder*, referring to online role-playing games. Authors such as Echeburúa (2012) have argued that while Internet and ICT-related disorders cannot be diagnosed, ever-increasing numbers of adolescents may require psychological treatment to combat excessive use.

Following Šmahel and Blinka (2012), this study uses the term “excessive ICT use” to refer not only to time spent on ICT use but also the impact of this. These authors argue that this term is frequently associated with determining pathological extensive usage of ICTs. Therefore excessive presence online is often defined by components used for determining other kinds of addictive behavior, such as mood change, conflicts and tolerance. According their findings, excessive use may lead to social, mental and physical impairment of adolescents. On the same line, Viñas (2009) ICT use is deemed excessive when the number of hours of use affects the correct development of the adolescent’s everyday life. So this term is used when the number of hours of use affects adolescents leading a normal daily life (Castellana et al., 2007; Viñas, 2009), but not only in terms of the time invested in this use but also in the impact that it causes in personal and social areas of adolescent life (Šmahel et al., 2012).

We do not use the term “addiction” in this study, thus avoiding the medical debate as to whether addictions to technologies can be diagnosed or not. All in all, whether diagnostic criteria for addiction to technology can be established or not, the rapid growth in access to ICTs increases problematic use, above all in adolescents, and is something that should be further researched. It is also necessary to teach responsible use of such technologies.

Certain personality traits have been considered to be determiners of an addictive personality (Nakken, 2013). Takao et al. (2009) stated that people with certain personality traits may be more susceptible to developing an addiction to technologies. Following the example of previous research (Schou et al., 2013; Kuss et al., 2014), this study takes the Five-Factor Personality Model, also known as “the Big Five” (Costa and McCrae, 1992), as a basis to explore which personality variables form the personality profile of excessive ICT users. According to this model, personality is formed by neuroticism, extraversion, openness, agreeableness and conscientiousness.

Profiles with high **neuroticism**, have been related to a problematic use of Internet (Öztürk and Özmen, 2011) and social networks (Marino et al., 2016). Recent studies have shown that high scores in this area may be associated with Internet addiction (Kuss et al., 2013), increasing the risk of developing addictive behavior (Wölfling et al., 2010). According to Amichai-Hamburger et al. (2002), those who score low in **extraversion** are more liable to use ICTs to connect to social networks as a way of satisfying their communication needs. Specifically, some studies define the profile of adolescents with problematic Internet use through the following: dissatisfaction with life; limited group cohesion and family support; a propensity to introversion; negative thoughts; discomfort with social relations; and identity conflicts (Echeburúa and de Corral, 2010; Kuss et al., 2013; Gómez et al., 2014). Moreover, **openness** has been linked to frequency of communication media usage (Correa et al., 2010). Schou et al. (2013) concluded that high scores in this dimension may prove to be a factor protecting against addictive behavior. While some studies relate problematic Internet use to high scores in **agreeableness** (Kuss et al., 2013), others conclude that this minimizes the risk of suffering from Internet addiction (Meerkerk et al., 2009). As for **conscientiousness**, Schou et al. (2013) conclude that conscientiousness may act as a protecting factor against such behavioral addictions and Wang et al. (2015) found that it is negatively associated with addictive behavior. As well as these 5 large-scale factors, much research has explored the relationship between **impulsiveness** (a facet of the neuroticism factor) and excessive ICT use. This has been linked to addictive behavior and difficulties in controlling those impulses that characterize addictions (Lee et al., 2012). Impulsive profiles are common in adolescents, who are great seekers of new sensations and instant rewards (Echeburúa et al., 2009). Previous research shows that those profiles with high levels of impulsiveness are more likely to develop excessive Internet use (Cao et al., 2007) and mobile phone use (Billieux et al., 2010).

Technologies provide a context of constantly gratifying stimulants that, as long as they are used responsibly, may provide adolescents with well-being. While some studies relate Internet use to aspects that may contribute to greater subjective well-being (Valkenburg and Peter, 2009; Malo et al., 2012), others suggest that this may, in fact, lessen it (Meerkerk et al., 2009; Akın, 2012). Excessive use is thought to affect well-being, causing anxiety and other psycho-pathological symptoms, as well as health, educational and social problems (Billieux et al., 2015). Some adolescents seek acceptance or social validation through social networks, and this affects their well-being and self-esteem (Jackson et al., 2010). According to Aydin and Volkan (2011), adolescents with low self-esteem dedicate more time to social network use. Yang and Tung (2007) and Bahrainian et al. (2014) related low levels of self-esteem with the presence of Internet addiction symptoms in adolescents. For Echeburúa (2012), self-esteem acts as a factor of protection against ICT addictions.

Creating and keeping a network of friends is considered vital to development during adolescence (Manago et al., 2012). The rapid growth of social networks has led many adolescents to use a range of technological tools to connect with their friends, create and strengthen interpersonal relationships, support others and receive social support, and cultivate emotional ties (Lenhart et al., 2010). Valkenburg and Peter (2009) argue that Internet use has a positive effect on interpersonal relationships between adolescents. While Echeburúa (2012) concluded that a healthy social environment and family support are protective factors against addiction to new technologies and social networks, family conflicts arising from irresponsible ICT use are ever more frequent. De Leo and Wulfert (2013) concluded that excessive ICT use may lead to an increase in family conflicts.

Family communication, parental control and supervision of online behavior have been considered to be protective factors against Internet addiction, along with family cohesion (Lin et al., 2009). Nonetheless, some research shows that 62% of those aged 15 and 16, surf the Internet without their parents having set limits (Sureda et al., 2010). Families play an important role in fomenting the opportunities that ICTs offer and minimizing their risks. A permissive or unstructured family environment with inexistent or inconsistent rules and a lack of consistency between the mother and father does not aid in forming a group of healthy behavior patterns; neither does it provide adequate self-control or emotional support (Echeburúa and Requesens, 2012). Although parental mediation of ICTs reduces the risk of developing addictive and risk behaviors (Chang et al., 2015), the lack of awareness shown by some parents regarding the risks associated with excessive ICT use, along with a lack of supervision, lead to unlimited access and greater frequency of use and have been linked to irresponsible use and addictive behavior. Sureda et al. (2010) concluded that parents needed to be made media literate, this would provide them with the knowledge needed to accompany their children in responsible use. According to Mayorgas (2009), giving children an example of healthy behavior promotes responsible behavior, which in turn counteracts addictive behavior. The great importance of the example given by parents in ICT use should also be highlighted, as parental ICT habits predict those of their offspring (Connell et al., 2015; Hiniker et al., 2016).

Although aforementioned studies show interesting findings, very few studies have investigated personality traits and social context factors simultaneously in the same investigation. The motivation of the study underlies in the evident lack of consensus in the scientific community to conceptualize if the excessive use of ICT can be considered an addictive disorder of the same nature as behavioral addictions. The excessive use of ICT among adolescents and their relationship with personality variables, among other individual variables, and social context, draws a complex frame, with multiple factors and relationships between them. Although, authors such as Marino et al. (2016) emphasize the importance of analyzing these variables together, the studies that evaluate them separately are more common. This paper aims to explore the set of variables in order to have a more plural view of the complexity of this psychological reality. Thereby, this study has the novelty of exploring both variables together. This study is conducted on adolescents’ psychological profile, personality social and context factors, to explore the impact of excessive use of ICTs in this population. Understanding excessive ICT use not only in terms of the time invested in this use but also in the impact that it causes, this study aims to explore the psychosocial profile of those adolescents who self-reported an excessive ICT use. Specifically, we aim to analyze: the socio-demographic characteristics of the group of adolescents identified as having excessive ICT use; the personality and social context variables that comprise the profile of self-reported excessive users; and which variables best predict excessive self-reported ICT use in the age group researched.

## Materials and Methods

### Design

This is a cross-sectional descriptive study. The multi-stage cluster sampling technique was used to define the sample.

### Participants

A random sample was chosen (n = 1,218) using the multi-stage cluster sample technique. This type of sampling was selected because it allowed us to obtain a large sample of subjects with relatively homogeneous characteristics and therefore, it would give us the possibility to explore the values of the variables object of our investigation from the creation of subgroups with a sufficient number of subjects. The study population comprised 11 to 18-year-old students (M = 14.42, SD = 1.78) at secondary compulsory education, baccalaureate, and professional training schools in the Alt Empordà region (Girona, Spain) (Table 1). The population comprised 5,365 students, 50.58% of whom were boys. 84.53% of the schools were state-run. In Spain compulsory secondary education starts in the first year of ESO when the student would be between 11 and 12 years old (equivalent to Year 7 in the United Kingdom education system) and finish in the fourth year of ESO. Baccalaureate is the university entry level course which lasts two years (equivalent to a 2-year A level course in the United Kingdom).

**Table 1:** Socio-demographic characteristics of sample.

Characteristics		n	%
Gender			
	Male	530	48.1
	Female	572	51.9
	Total	1.102	100
Age
	11–12	185	16.8
	13	220	20
	14	171	15.5
	15	174	15.8
	16	187	17.0
	17–18	165	15.0
Type of centre	State-run	1.009	91.56
	Semi-private	93	8.44
Academic year^∗^	1st ESO	201	18.2
	2nd ESO	224	20.3
	3rd ESO	191	17.3
	4th ESO	177	16.1
	1st BAT	167	15.2
	2nd BAT	111	10.1
	Professional training cycle	31	2.8


*^∗^*ESO* is compulsory secondary education. A student in the first year of ESO would be between 11 and 12 years old (equivalent to Year 7 in the United Kingdom education system). *BAT* is the university entry level course (equivalent to a 2-year A level course in the United Kingdom).*

In a first stage the educational center was selected and after that, we selected one class of each academic course. Using this sampling technique, a random sample was selected (n = 1218).The final sample was of 1,102 students (90.48% participation). The remaining students were discarded because they were outside the age range or they did not finish answering the questionnaire. We also exclude the questionnaires from the adolescents who copied the answers from their classmates and those who did not show interest in answering the questionnaire. Students from 6 centers took part, 1,009 (91.56%) from state-run centers, and 93 (8.44%) from a semi-private center. The sample is composed of 530 boys (48.1%) and 572 girls (51.9%).

### Measures

A protocol was drawn up including standardized scales and *ad hoc* items. Students were first asked to fill in the name of their school, their academic year, age, date of birth, and gender; these data were then used as grouping variables.

The scales used to assess personality and social context were:

- **Personal Well-being Index** (PWI) (Cummins et al., 2003): The PWI consists of 7 items that assess the degree of satisfaction with the following aspects of life: standard of living; health; achievements in life; personal relationships; perception of personal security; security with the groups they belong to; and future security. Each item was assessed on an 11-point scale, from 0 (*Totally unsatisfied)* to 10 (*Totally satisfied*). The translation of the original version of the domains included in the PWI into Catalan and its back-translation from Catalan to English was the work of Casas et al. (2008). The internal consistency of the PWI in the present study was good (α = 0.86).
- **Multidimensional Scale of Self Concept** (AF5) (García and Musitu, 2001): The Catalan-adapted version was applied (Malo et al., 2014), consisting of 30 items contemplating the 5 self-concept dimensions proposed by the original authors (academic/professional; social; family; physical; and emotional). An 11-point scale was used, from 0 (“*Never*”) to 10 (“*Always*”).

The psychometric properties of this scale are very good and similar to the original version. The internal consistency found in this study ranges from 0.75 (social) to 0.91 (academic).

- **Social Support Appraisals** (SSA) (Vaux et al., 1986): The original scale consists of 23 items that explore the perception boys and girls have of the social support they receive from their family, friendships and others in general. This study has used 14 of the items, 7 referring to the family and 7 to friendships. The two dimensions have been calculated using the totals given to each, with responses ranging from 0 (*“Not at all”*) to 10 (*“Very clearly”*). The internal consistency of the *friends* dimension of SSA is 0.91, and that of *family 0*.92.
- **NEO Five Factor Inventory** (Costa and McCrae, 1992, 2004): reduced version of the NEO PI-R, which permits the assessment of five personality traits: *neuroticism; extraversion; openness to experience; agreeableness;* and *conscientiousness.* It consists of 60 items scored on a 0 to 4 point Likert scale (*0 = Totally disagree, 4 = Totally agree*). The internal consistency of the personality trait scales was acceptable, with a Cronbach alpha of 0.64 for neuroticism; 0.61 for extraversion; 0.62 for openness; 0.53 for agreeableness; and 0.69 for conscientiousness. The items from the NEO PI-R (Costa and McCrae, 2008) related to impulsiveness were added. The internal consistency of impulsiveness was good, displaying a Cronbach alpha of 0.74.

In order to examine ICT use, the following were used:

- **ICT use Self-classification scale** (Casas et al., 2007). A single-item scale in which subjects self-classify the type of consumer they are based on the following categories: *1: I never or hardly ever use it; 2: I’m a low consumer; 3: I’m an average consumer; 4: I’m a fairly high consumer; 5: I’m a very high consumer.* The same scale was used to ask adolescents about the kind of ICT users their parents and siblings are. Reliability cannot be evaluated because it is a single item scale and therefore Cronbach’s alpha cannot be obtained. Casas et al., 2007 to validate this single item scale made a correlation between the number of hours of the ICTs use and the self-categorization. In their study found a good congruence of answers (r = 457, p < 0.001).

An *ad hoc* questionnaire was added to ascertain frequency and type of ICT use, which included the following items:

- Frequency of use of mobile phone, tablet, computer, and videogames on a scale of 1-5 (*1: Never, 2: Rarely; 3: Often; 4: Very often; 5: Continually*).
- A dichotomous question (*yes/no*) regarding the existence of rules regarding ICT use.
- A polynomial question (multiple answers), where subjects mark whether ICT use has led to problems related to: *performance at school; relations with friends; relations with parents; relations with teachers; sports activities; reduction in leisure activities;* *I’ve used them excessively; I’ve received threats; I’ve lied or pretended to be someone else; I’ve made inappropriate comments; I’ve spent more money than I should have; I’ve visited sites with age-inappropriate content*.

### Procedure

This research is part of a broader study on the use of ICT in adolescents. In addition to explore adolescents personality and social context factors associated to self-reported excessive use of Information and Communication Technology, we also focused in the impact of social networking and the consequences of media multitasking.

This study was carried out in accordance with the recommendations of Education Department from the Autonomous Government of Catalonia. After obtaining permission from the Autonomous Government of Catalonia’s Education Department and the individual schools, the head teacher of each school was informed of the characteristics and aims of the research. In conducting the study we followed the ethical guidelines of the Helsinki Declaration, with written informed consent obtained by the participants’ parents and the school authorities. Schools and participants were guaranteed data confidentiality and anonymity. The data collection for this study was anonymous, voluntary and non-interventionist; therefore, in accordance with local legislation and national guidelines a full review and ethical approval were not required.

Once all scales were included on a single questionnaire, this was split into two parts in order to make it less tiring for participants. The questionnaire was administered in two one-hour sessions on different days during the 2016-2017 school years, and completed in the classroom in school time. Participants received specific, homogenous instructions regarding how the questionnaire should be answered. They were accompanied by instructors who had received prior training in the research to give any help or clarification.

### Statistical Analysis

Data were analyzed according to gender and school year using the Student’s t-test to compare means, and through the Chi-square test to compare proportions. Given the low number of 11 and 18-year-old students, and in order to adjust data to provide more homogeneous groups, 11 and 12-year-old students were grouped together, as were 17 and-18 year-olds.

Taking into account that excessive use of ICTs not only implies a high use but also interferes with the normal functioning of the subject, a group was created of those adolescents who use ICTs excessively. It should be noted that in addition to the self-categorization, other information has been taken into account to make excessive use if ICTs group. So, adolescents of this group affirmed that they have had problems because of the excessive use of ICTs. This fact implies that the adolescent is aware that his use of ICT has had a negative impact. In addition of the adolescents’ responses to self-classification scale (Casas et al., 2007) the frequency of self-informed use of ICT was taken into account.

The group comprises those who answered: “*5: I’m a very high consumer*” on the ICT use self-classification scale (Casas et al., 2007); “*4: Very often”* or *“5: Continually”* for the *ad hoc* ICT use frequency question on: *the mobile phone; tablet; computer;* and/or *videogames;* and those who marked “*I’ve used them excessively”* for the *ad hoc* polynomial question asking whether they had experienced any problems resulting from ICT use.

Finally, binary logistic regression was used to construct a model of those factors that best predict excessive ICT use.

All analyses were carried out using the SPSS, version 23.0 statistical package. The minimum level of statistical significance required in all tests was *p* < 0.05.

## Results

14.5% (n = 160) of the total sample were included in the group of self-reported excessive users. Of these, 49.4% (n = 79) were boys and 50.6% (n = 81) girls; no significant differences were noted between the groups (X^2^ (1, *N =* 1102) = 0.123, *p* = 0.726). 41.3% (n = 56) of the group of excessive users were 15 or 16. While 60% (n = 86) of self-reported excessive users were in the 3rd and 4th years of secondary school and the 1st year of the university entrance course, no significant differences were found between school years (X^2^ (1, *N =* 1102) = 10.053, *p* = 0.122) (Table 2).

**Table 2:** Percentage of the group of self-reported excessive ICT use according to gender and age.

	ICT self-reported excessive use group							
Variables	Yes (*n* = 160)		No (*n* = 942)					
	N	%^(∗)^	n	%	Total	%	χ^2^	P
Gender								
Male	79	49.4%	451	47.9%	530	48.1%	0.123	n.s.
Female	81	50.6%	264	52.1%	572	51.9%		
Age								
11–12	18	11.3%	150	15.9%	166	15.1%	8.546	0.129
13	27	16.9%	193	20.5%	220	20%		
14	26	16.3%	145	15.4%	171	15.5%		
15	32	20%	142	15.1%	174	15.8%		
16	34	21.3%	153	16.2%	187	17%		
17–18	23	14.1%	115	15.1%	142	12%		


*^(∗)^% of ICT self-reported excessive use group.*

### Personality and Excessive ICT Use

Scores in the group of self-reported excessive use of ICT in the five personality dimensions of the NEO Five Factor Inventory were higher in the *neuroticism* and *extraversion* dimensions and lower in those of *openness to experience, agreeableness* and *conscientiousness* than in the group that does not (see Table 3). If we look at impulsiveness, the average scores for self-reported excessive users are higher. These differences are statistically significant in: *neuroticism, agreeableness, conscientiousness and impulsivity*.

**Table 3:** Average scores in the 5 personality dimensions of the NEO-FFI and impulsiveness aspect of the NEO PI-R, depending on whether or not they form part of the self-reported excessive ICT use group.

Variables	Self-reported excessive ICT use							
	Yes		No					
	M	SD	M	SD	t_(gl)_	p	IC 95%	
Neuroticism	1.93	0.732	1.77	0.753	–2.490__(1100)_	0.013	–2.854	–0.033
Extraversion	2.28	0.824	2.22	0.719	–0.975__(1100)_	n.s.	–0.185	0.062
Openness	2.05	0.724	2.09	0.731	0.668__(1100)_	n.s.	–0.081	0.164
Agreeableness	2.09	0.740	2.27	0.674	3.120__(1100)_	0.002	0.068	0.297
Conscientiousness	1.88	0.762	2.19	0.689	5.092__(1100)_	< 0.001	0.187	0.422
Impulsiveness	2.10	0.637	0.709	3.185	–12.065__(1100)_	< 0.001	–1.617	–1.165


### Well-Being, Self-Concept and Excessive ICT Use

Average scores were lower in the group of self-reported excessive users (Table 4).

**Table 4:** Average scores for well-being and self-concept depending on whether they form part of the self-reported excessive ICT use group or not

Variables	Excessive ICT use							
	Yes		No					
	M	SD	M	SD	t_(gl)_	p	IC 95%	
PWI – Subjective well-being	76.72	13.942	80.50	14.190	3.121__(1100)_	0.002	1.402	6.151
AF5 – Family self-concept	7.45	2.023	8.149	1.749	4.091__(1100)_	< 0.001	0.360	1.030
AF5 – Academic self-concept	5.72	2.097	6.82	1.835	6.853__(1100)_	< 0.001	0.784	1.414
AF5 – Social self-concept	7.30	1.526	7.282	1.651	–0.159__(1100)_	n.s.	–0.296	0.252
AF5 – Emotional self-concept	4.90	1.999	5.33	1.96	2.545__(1100)_	< 0.011	0.098	0.756
AF5 – Physical self-concept	6.10	2.140	6.75	1.851	3.583__(1100)_	< 0.001	0.289	0.998


In all components bar social self-concept, there are significant differences between those who use ICTs excessively and those who do not. Adolescents in the group of self-reported excessive users score significantly lower in the family; academic; emotional; and physical components (Table 4).

### Social Context and Excessive ICT Use

When examining results for the dimension on perception of social support from friends, we note that the scores for those who self-reported excessive use of ICTs (M = 7.91, SD = 1.684) are very similar to the ones for those who do not (M = 7.90, SD = 1.702). In contrast, there are statistically significant differences between the groups when it comes to the perception of support from the family (*t*(1100) = 1.977 *p* = 0.048), with scores among the group excessive use of ICTs lower (M = 8.11, SD = 1.764) than among those who do not (M = 8.40, SD = 1.702).

When the group of self-reported excessive use of ICTs was asked about their perception of their parents’ ICT use, higher scores were obtained when defining the parents as *fairly* or *very high* ICT users. While 17.7% of adolescents whose ICT use is not excessive said of their mother “*I consider her a fairly high consumer*”, and 3.2% “*she is a very high consumer*”, the percentages are higher among the group of excessive users: 32.1% and 9.4%, respectively. As for the father, while 11% of the group who do not self-reported an excessive use respond “*I consider him a fairly high consumer*”, and 2.9% “*he is a very high consumer*”, the respective percentages for the group that self-reported an excessive use are 19.7% and 9.2%. These differences are statistically significant, for both the mother’s use (X^2^ (1, *N =* 977) = 36.240, *p* < 0.001) and that of the father (X^2^ (1, *N =* 949) = 32.747, *p* < 0.001).

In order to explore differences in the categorization of sibling ICT use, a variable was created calculating the mean of such use. Average ICT use among siblings of the group that self-reported an excessive use of ICTs (M = 3.40, SD = 1.7) is higher than among siblings of people who do not belong to the group (M = 2.58, SD = 1.71), these being statistically significant differences (*t*(852) = -6.539 *p* < 0.001).

There are significant differences in the existence of rules on ICT use between the group that self-reported an excessive use and the group that does not. 69% (n = 109) of those in the former group say there are no rules, a percentage that falls to 58.1% for the latter group (X^2^ (1, *N =* 974) = 6.544, *p* = 0.011).

### Factors That Predict Excessive ICT Use

In order to identify those variables that predict self-reported excessive ICT use among adolescents, a binary logistic regression was carried out using the stepwise method. The group making excessive use was considered to be a dependent variable, and the dimensions of personality, social context and sex covariates. As can be seen from Table 5, the model enables the correct classification of 83.1%, (n = 945) of participants; 78.8% of the group that does not make excessive use of ICTs and 58.7% of the group of excessive users. Nagelkerke’s *R^2^* shows that the model explains 18.9% of the variability (Nagelkerke *R2* = 0.189).

**Table 5:** Binary logistical regression of personality factors, social context variables and gender in self-reported excessive ICT use.

Steps	Variables	B	E.T	Wald	gl	P	OR	IC 95%	
Step 6(g)	Maternal ICT use	0.407	0.107	14.531	1	< 0.001	1.502	1.219	1.852
	Paternal ICT use	0.274	0.100	7.562	1	0.006	1.316	1.082	1.599
	Sibling ICT use	0.139	0.067	4.370	1	0.037	1.150	1.009	1.310
	AF5 Academic	–0.216	0.058	13.709	1	< 0.001	0.805	0.718	0.903
	Impulsiveness	0.099	0.031	10.375	1	0.001	1.104	1.039	1.172
	Conscientiousness	–0.400	0.173	5.364	1	0.021	0.670	0.478	0.940
	Constant	–3.439	0.808	18.128	1	< 0.001	0.032		


Variables that predict excessive ICT use are, in order of weight: perception of mothers ICT use; responsability; perception of fathers ICT use; academic self-concept; perception of sibling ICT use; and impulsiveness. The factors protecting against excessive ICT use are the perception of parental and sibling ICT use and academic self-concept. Risk factors are impulsiveness and responsibility.

## Discussion

Our results confirm an elevated prevalence of self-reported excessive ICT users, 14.5% of the sample studied. This percentage is similar to that seen in other studies (Durkee et al., 2012), which situate problematic use of the Internet at around 13.5%. With regard to socio-demographic variables, and in line with research which concludes that ICT use begins at an ever-earlier age (Fernández-Montalvo et al., 2015), it should be highlighted that 11.3% of the group of excessive users were 11 or 12 years old. While previous research shows an age-based increase in ICT use (Devís-Devís et al., 2009), our study found no differences between the age of users. Regarding gender, the percentage of boys and girls who self-reported an excessive use of ICTs was similar. These data differ from research that has found problematic Internet use to be significantly higher among girls (Durkee et al., 2012; Rial et al., 2015).

A psychological profile associated with self-reported excessive use of ICT is provided by the responses given to the various items of the NEO-FFI and in the impulsiveness factor of the NEO PI-R. Those adolescents whose ICT use is problematic describe themselves as emotionally unstable, impulsive and not very conscientious and agreeable. Our results coincide with those of Wang et al. (2015), where conscientiousness is negatively associated with addictive behavior and with Kuss et al. (2014) where problematic Internet use is related to high scores in agreeableness. Along the lines of previous research into problematic use of the Internet (Öztürk and Özmen, 2011), and of social networks (Marino et al., 2016), our study links excessive ICT use with higher scores in neuroticism. The impulsive profile predominates among those young people who self-reported an excessive use of ICTs, matching earlier studies which link high levels of impulsiveness with excessive mobile phone use (Billieux et al., 2010) and problematic Internet use (Cao et al., 2007). These results are similar to those of earlier research, which show that the psychological profile of those with high Internet use is one where negative emotions predominate (Viñas, 2009).

Regarding the social context variables examined the index of subjective well-being and family social support is lower among those who make excessive use of ICTs than among other young people. Previous research also concludes that excessive ICT use reduces well-being (Meerkerk et al., 2009; Akın, 2012) and self-esteem (Yang and Tung, 2007; Bahrainian et al., 2014). Low scores in family social support may be explained by the intrusive nature of the technology and its potential to interrupt or interfere with family life. According with De Leo and Wulfert (2013) and McDaniel and Coyne (2016), the omnipresence of technology may interfere in human relationships, and that this may affect family social support. Regarding the self-assessments of those adolescents who form part of the group of self-reported excessive users, we note that that they are lower than among those not in the group for the following facets: quality of performance in their role as students; emotional state, and their responses in certain situations, with a degree of commitment and implication in their daily lives; their involvement, participation and integration in the family; and physical appearance and condition. Given the close link between the terms *self-concept* and *self-esteem*, the results lead us to conclude that adolescents who use ICTs excessively show lower levels of self-esteem than those who do not. These results would imply that adolescents with lower self-esteem may be more vulnerable to excessive ICT use. This is also confirmed by certain other studies (Bahrainian et al., 2014) which show that self-esteem is one of the best predictors of addictive Internet behavior.

Consistent with previous research, there seems to be little parental mediation in adolescent ICT use. 58.1 % of participants said there were no rules regarding ICT use. This percentage is similar to that of other research, which places it between 53% and 62% depending on the child’s age (Sureda et al., 2010). As family mediation is a determining factor in children’s ICT use and a lack of supervision is linked to addictive behavior (Chang et al., 2015), rules regarding ICT use should be promoted. In order to achieve greater parental involvement in establishing such rules, we believe that parents need to be made more media literate, as has been highlighted in earlier research (Sureda et al., 2010). The results obtained regarding perception of ICT use among parents and siblings show that adolescents who form part of the group of excessive users perceive their parents to be higher consumers of ICTs than those of the other group. These results lead us to conclude that the frequency with which children use ICTs is influenced by their parents’ example. In the same vein, research into Internet use concludes that parental habits influence those of their children (Connell et al., 2015; Hiniker et al., 2016).

The ultimate aim of our research has therefore been to assess those variables that best predict self- reported excessive ICT use at this age. When all personality and social context variables are included, and through logistical regression analysis, six factors identifying a risk of excessive ICT use have been identified: perception of mother’s ICT use; conscientiousness; perception of father’s ICT use; academic self-concept; perception of sibling’s ICT use; and impulsiveness. These factors enable us to establish a predictive model of excessive ICT users. Perceiving that parents and siblings are high ICT consumers all increase the risk of excessive ICT use, the risk factor that best predicts excessive use is the perception of mother ICT use. These results suggest that parents’ ICT use predicts their children’s habits (He et al., 2010). The family plays a central role in the transmission of values, and it is for this reason that the prevention of excessive use must include parents as central agents in intervention. To achieve this, families must be literate in the digital world; a lack of knowledge can lead parents to adopt inadequate positions when fomenting ICT use, such as a lack of supervision. We agree with Echeburúa (2012) that ICT use involves parents and adolescents sharing responsibility. The latter should aid their parents to become familiar with the use of technologies, while parents should accompany their ICT use of their children. The family, therefore, plays a fundamental role in minimizing risks associated with ICT use.

Referring to factors of protection, the results show that, while having high scores in responsibility dimension and low scores in impulsivity. These discoveries coincide with previous research concluding that impulsive adolescents are more prone to developing problematic Internet use (Billieux et al., 2010).

This study has provided us with a psychosocial profile of adolescents who self-reported an excessive use of ICT, which may be of help in preventing such use. The presence of specific personality factors such as impulsiveness, of factors related to social context, such as high parental and sibling use, all indicate a greater vulnerability and may serve as indicators for parents, teachers and healthcare professionals to intervene and prevent excessive ICT use and other serious psychological problems related. So, for the detection of excessive use of ICT, professionals must analyze individual differences and contextual realities of the adolescents.

In the same line as Armayones (2016), it is essential that health professionals are able to identify those who have a greater predisposition to develop disorders derived from the use of ICT, although they have not yet expressed problems. It is important to know how to distinguish those problems derived exclusively from the use of ICT, which are actually the result of another psychological disorder. The type of use that is given to ICT and the excessive frequency can affect various facets of life and develop a disorder. However, the presence of psychological disorders (such as anxiety, depression or personality), problems of self-esteem or deficits in social skills can lead to excessive use of ICT. In the first case, the problem that gives rise to excessive use could be related to the frequency or access to inappropriate content. In contrast, in the second case, excessive use would become a type of coping strategy for another problem or disorder of the subject. Therefore, for the detection and approach of the excessive use of ICT it will be essential to carry out an individualized analysis of the personal and contextual reality of each adolescent.

These discoveries have provided a psychosocial profile but future research exploring both factors can also be helpful to solidify these results. The results of this study would allow us to develop new measures for the evaluation of excessive use of ICT to more deeply analyze psychological and social context variables of the adolescents. Our findings can also help to create interventions that take into account the regulation of certain traits of personality, as impulsivity, and the responsible use in family context of the adolescents. Another direction for future research delves further into focusing on identifying the psychological and social profile of adolescents who make responsible use with the aim of creating models of positive socialization in the use of ICT.

Taking into account that the profile of excessive users group is comprised of the combination of personality and the closest social context, we would like to emphasize the need of engaging in further investigations that examine both variables. Our results allow us to propose some feasible interventions with the aim to prevent excessive ICT use among adolescents. Considering impulsiveness as a risk factor and conscientiousness dimension as a protective factor against excessive ICT use, we suggest specific actions to promote the regulation of impulsivity trait. For instance, mindfulness programs with the goal to promote adolescents’ full conscientiousness. The results point out parents and siblings consumption of ICT acts as a risk factor. That is the reason why we propose interventions with the family context. Following Gómez et al. (2017), it is necessary to include the immediate social context, family and educators, in the interventions to promote responsible use. Our intervention proposal coincide with Sureda et al. (2010), showing the need for awareness and provision of knowledge and skills to engage parents to assume their responsibilities in the use of ICT of adolescents. Psychoeducation program which include specific actions to: a) promote adolescents’ self-conscientiousness of the time spent on ICT use, 2) show the risks of excessive use of ICT, 3) engage parents and educators to work together to promote responsible use among adolescents, 4) emphasize the importance of rules and standards set, not only with temporary restrictions, and the participations of the adolescents in the decision of these rules.

We must be aware that children from birth to adolescence observe adults at home, imitate behaviors and repeat actions. If our attitude is prohibitive, they will see ICT as negative or harmful tools, and they will not develop the ability to use and criticize the media. If our attitude is carefree, very permissive, or irresponsible, we will not help them know how to manage the use of these tools as they grow. Making an appropriate use of home-based technologies implies not using them just as a babysitter to keep children entertained, but rather to have a proactive attitude of how and how we can use them. Playing, learning, expressing, communicating and creating are the potentialities of these resources. And that is possible implies setting activities and timings to be able to use them. It also implies the need to accompany the adolescents in their use as parents and educators. Not forgetting that each adolescent is different, has different interests, different learning rates and special needs in each case, it is possible to establish general guidelines. From early childhood to adolescence, we have to think that at home we have to take control of children’s technology. We must use its potential as a game and learning tool, selecting good resources, establishing habits and rules of use that benefit the development of children and actively participating in the use of ICTs at their side. It is important to bring awareness to the parents that technology is much more than a device that we have to regulate. ICTs can be tools for game and fun, for learning and development. Taking informed decisions about when, how, what, etc. to use ICT will help them to establish healthy habits of ICT usage at home.

There are some limits to this study. Firstly, the sample comprises adolescents of the same age range and geographical location; participants from other regions would be needed for results to be extrapolated. The sample, while representative of a region and age range, does not permit generalization to other population groups. Even though the sample has been chosen randomly, it has been stratified by center and is representative in different socioeconomic levels; we cannot generalize the results to all adolescent population further than the geographical area studied. Some cultural and social differences could exist compared with other areas. A further limitation is the self-reporting nature of the instrument, as participants may show a bias toward social desirability; however, we have attempted to minimize this through the guarantee of anonymity and data confidentiality.

Another aspect to consider is the limitation of identifying adolescents who made an excessive use of ICTs based on self-reports administered in an academic environment. In general terms, teens are good informants although in the collective context it is likely to be bias. Therefore, self-reports should be supplemented with objective measures such as structured clinical interview (Beard, 2005). In future research, the administration of questionnaires could be done in groups with less number of participants than the regular group class.

Future research could include qualitative methodology; discussion groups, for example, in order to increase data validity and further explore the knowledge gained from questionnaires. Since this is a cross-sectional study, we cannot determine causative relations, so future research could include longitudinal perspectives to gain greater depth into the prediction of excessive ICT use.

## Author Contributions

MM-P, has designed the research work and the questionnaire, collected the data and contributed to the analyses and interpretation of the findings and was accountable for all aspects of this work. FP has designed the research work, conduced the data analyses and interpretation of the findings and contributed to writing methodology and results sections of the paper. SC has designed the research work, collected data and contributed to the interpretation of the findings and contributed to writing the introduction and discussion sections of the paper. All authors listed had made a substantial contribution to the work and have drafted and revised the work and approved the submitted version.

## Conflict of Interest Statement

The authors declare that the research was conducted in the absence of any commercial or financial relationships that could be construed as a potential conflict of interest.
